# ZNF692 promotes the migration and response to immunotherapy of clear cell renal cell carcinoma cells by targeting metabolic pathway

**DOI:** 10.1007/s12672-024-01005-0

**Published:** 2024-05-12

**Authors:** Yuming Liu, Dehua Zeng, Yunzhen Gao

**Affiliations:** 1 Department of Anesthesiology, MengChao Hepatobiliary Hospital of Fujian Medical University, Fuzhou, 350025 China; 2 The Institute of Psychiatry and Neurology Medicine, Xinxiang Medical University, 601 Jinsui Road, Xinxiang, 453003 China; 3 Department of Pathology, The 900, Hospital of Joint Logistic Support Force, PLA, Fuzhou, 350025 China

**Keywords:** ZNF692, Clear cell renal carcinoma, Zinc finger protein, Migration, Immune checkpoint blockade

## Abstract

Clear cell renal cell carcinoma (ccRCC), with high mortality and poor prognosis, is the most common type of renal malignancy. It is necessary to identify new biomarkers that can serve as indicators for the detection of ccRCC at its early stages. In this study, we analyzed the role of classical zinc finger protein 692 (ZNF692) in ccRCC using datasets from The Cancer Genome Atlas (TCGA) and Single Cell Portal and immunohistochemical (IHC) staining of a tissue-microarray, and analyzed the function of ZNF692 in ccRCC cells. The analyses indicated that ZNF692 was upregulated in ccRCC samples compared with normal or paracancerous control samples (P < 0.001) and that the expression of this gene was linked to poor overall survival (HR = 2.1, P < 0.0001). The knockdown of ZNF692 inhibited the proliferation and migration of ccRCC cells by target GTPase-activating protein (SH3 domain)-binding protein 2 (G3BP2), and transmembrane 9 superfamily member 2 (TM9SF2)). T, B, proximal, and collecting tubule cells are the dominant cell types in normal kidney tissue where ZNF692 is expressed. In addition, immune checkpoint blockade (ICB) therapy dramatically changed the expression patterns of ZNF692. Collectively, these data indicate that ZNF692 may serve as prognosis, and as a potential indicator of the response to ICB therapy, a possibility needs to be verified by a case‒control study.

**Supplementary Information:**

The online version contains supplementary material available at 10.1007/s12672-024-01005-0.

## Introduction

Renal cell carcinoma (RCC) is the most common type of renal malignancy, accounting for 90–95% of all renal cancers and 2–3% of all cancers [1]. Over the last 10 years, there has been an increase of approximately 20% in the incidence of RCC, especially in industrialized countries [2]. Clear cell renal cell carcinoma (ccRCC), originating from proximal tubule cells, is the most common histologic subtype of RCC, especially advanced RCC (accounting for 60–70% and 90% of cases, respectively) [3]. Due to its largely asymptomatic nature and distinguishable clinicopathological risk factors, ccRCC is rarely detected in the early stage [4]. In addition, up to one-third of patients with ccRCC develop metastasis or present with metastasis at the time of diagnosis [5]. Unfortunately, the disease at this stage is almost always fatal. Although steady progress has been made in delineating the molecular characteristics to explain the initiation and progression of this lethal disease [6], it is an urgent need to mine ccRCC datasets to identify novel potential molecules that can be used to detect ccRCC in early stages.

Classical zinc finger proteins (ZNFs), constituting a large family of sequence-specific transcription factors, are encoded by 2% of human genes [7]. Over the last few decades, it has been reported that aberrant expression of ZNFs is involved in the initiation and progression of tumors in various malignancies [8–10]. ZNF692, located on chromosome 1q44, is a key transcription factor that binds to the promoter regions of key genes regulating tumor progression [11]. Recent reports have shown that ZNF692 is linked to the recurrence of Wilms’ tumor [12]^,^ the prognosis and proliferation of ccRCC [13, 14], and the aggressiveness of lung adenocarcinoma (LUAD) [15] and that it promotes the proliferation and invasion of cervical cancer cells [16]. In addition, ZNF692 may serve as a potential biomarker associated with immune infiltration in hepatocellular carcinoma [17]. Though, Wang et al reported that ZNF692 promoted the proliferation of ccRCC cells by targets IRF4 and FLT4 [14], however, the role of ZNF692 in ccRCC is largely unknown.

In this study, we found that ZNF692 was upregulated in ccRCC and that its expression was increased in a stage-specific manner. The expression of ZNF692 was associated with poor overall survival. In addition, ZNF692 promoted the proliferation and migration of ccRCC cells by targeting G3BP2 and TM9SF2. Moreover, ZNF692 was expressed mainly in proximal tubule cells, collecting duct cells, and T and B cells in normal kidney tissue. However, upon exposure to immune checkpoint blockade (ICB) agents, a portion of ZNF692-positive tumor cells (referred to as tumor program 1 cluster (TP1) cells) in advanced ccRCC patients responded to ICB therapy. These data indicate that ZNF692 might be involved in the migration and ICB response of ccRCC cells.

## Materials/patients and methods

### Patient and tumor samples

Fifty-eight paraffin-embedded cancer and paracancerous samples were collected between January 2019 and December 2022 at The MengChao Hepatobiliary Hospital of Fujian Medical University. The samples were collected based on specific inclusion and exclusion criteria. The inclusion criteria consisted of primary clear cell renal cell carcinoma (ccRCC) cases that had not undergone any treatment prior to surgery. Conversely, the exclusion criteria encompassed other types of kidney carcinoma, non-primary ccRCC cases, and individuals who had received any form of interventional therapy, such as radiotherapy or chemotherapy. All patient is still alive at the time of writing the manuscript. All samples were obtained from patients with primary ccRCC aged 32 to 84 years. The TNM stage of the patients ranged from 1 to 4, with stage 2 being the most common stage (Table SI). The study was approved by the Institutional Review Board of MengChao Hepatobiliary Hospital of Fujian Medical University for the use of patient samples. Informed consent form was received from all participants before surgery in this study. Pathologic and clinical diagnoses of ccRCC were made using the diagnostic criteria of the International Society of Urological Pathology (ISUP) consensus. All methods were performed in accordance with the relevant guidelines and regulations of the Institutional Review Board of MengChao Hepatobiliary Hospital of Fujian Medical University.

### Gene expression analysis

To explore the expression profile of ZNF692 in ccRCC, we queried The University of Alabama at Birmingham Cancer data analysis Portal (UALCAN, http://ualcan.path.uab.edu/analysis.html) to assess the expression levels of ZNF692 in ccRCC in a dataset from The Cancer Genome Atlas (TCGA) using normal kidney tissue as a control [18].The input gene symbol "ZNF692" was utilized to query its expression within the TCGA dataset "Kidney renal clear cell carcinoma (KIRC)". To compare the expression of ZNF692 between paired tumor and adjacent normal tissues, we queried the Tumor Node Metastasis (TNM) plotter (https://www.tnmplot.com) website [19]. Using “ZNF692” as keyword, we obtained mRNA expression data for ZNF692 in tumor and adjacent normal tissues and those data were analyzed by the Mann‒Whitney test.

### Single-cell RNA sequencing data analysis

To identify the expression pattern of ZNF692 in normal kidney tissue, we queried the scRNA section of the Human Protein Atlas website (https://www.proteinatlas.org) using ZNF692 as keyword [20]. To explore the role of ZNF692 in ccRCC, we queried Single Cell Portal (https://singlecell.broadinstitute.org/single_cell) using keywords “kidney” and “cancer” to search related studies. After identified the studies, we explored the expression pattern of ZNF692 in different cell types exposed to ICB agents using the dataset from the study by Kevin Bi et al [21] using “gene explore” tools and “ZNF692” as keyword.

### Overall survival analysis

To determine the prognostic value of ZNF692 expression in ccRCC, the web tool Gene Expression Profiling Interactive Analysis (GEPIA, https:// http://gepia.cancer-pku.cn/detail.php) was used to visualize the correlation between ZNF692 expression and the overall survival (OS) of ccRCC patients, with the median expression level as the final stratification cutoff [22]. We evaluated the potential prognostic value of ZNF692 using the least absolute shrinkage and selection operator (LASSO)–Cox regression method. The receiver operating characteristic (ROC) curve was plotted using the “survival” package in R.

### Gene Ontology and pathway analyses

To explore the possible functional mechanism of ZNF693 in ccRCC, we analyzed genes coexpressed with ZNF692 and performed Gene Ontology (GO) enrichment analysis using LinkedOmics (http://www.linkedomics.org) [23]. The gene associated with ZNF692 was obtained using RNA-seq data from the "sample cohort: TCGA_KIRC" by employing the "LinkFinder" tools. Subsequently, GO analysis was conducted using the ZNF692 associated genes as input through the utilization of GSEA tools. To explore the function of ZNF692 in patients treated with ICB agents, we performed correlation analysis using the “corrr” package in R to identify genes that were coexpressed with ZNF692 in tumor cells with or without exposure to ICB agents. GO enrichment analysis was performed using Metascape (http://metascape.org/gp/index.html#/main/step1) [24].

### Tissue microarray and immunohistochemical (IHC) staining

The tissue microarray, containing 29 pairs of primary ccRCC and paracancerous samples, was constructed in house. Briefly, small punches from 29 paraffin-embedded (donor) tissue blocks were obtained with a manual tissue arrayer, and those tissue cores were transferred into a positionally encoded array in a recipient paraffin block. After being sealed with paraffin, the tissue blocks were fixed on a tissue slicer and were sliced into serial sections with a 5 μm thickness. After deparaffinization, IHC staining was performed using an anti-ZNF692 antibody (Bioss, Beijing, China, Cat: bs-4360R) and horseradishperoxidase (HRP) conjugated second antibody (Beyotime, Beijing, China, Cat: A0208). After IHC staining, the sections were scanned and imaged using a scanner (Motic, Xiamen, China). The first sample in the tissue microarray was used as a reference for normalization among sections. The IHCF results were presented as two parameters: the signal intensity (scored from 0 to 4) and the percent of positively stained cells (positively stained cells/total cells per field of view, scored from 0 to 4. A score of 0 indicated the absence of staining, 1 indicated that the percentage of positively stained cells was less than 25%, 2 indicated that the percentage of positively stained cells was between 25 and 50%, 3 indicated that the percentage of positively stained cells was between 50 and 75% and 4 indicated that the percentage of positively stained cells was more than 75%). The total IHC score was calculated by summing the scores of the signal intensity score and the percentage of positively stained cells.

### Cell culture and transfection

The ccRCC cell lines Caki-1 and 786–0 were gifts from Professor Hongbo Zhao (Kunming Medical University). The cell lines were maintained in Dulbecco’s modified Eagle’s medium (DMEM, HyClone, Utah, USA) supplemented with 10% fetal bovine serum (PAN-biotech, Adenbach, Germany), 100 U/ml penicillin and 100 µg/ml streptomycin (Gibco, New York, USA) at 37 ℃ with 5% CO_2_. All those cell lines were mycoplasma free as tested by PCR methods. For transfection, 2 × 10^5^ cells per well were seeded in a 6-well plate and cultured for 12–18 h. When the cells were 50–70% confluent (for siRNA transfection) or 80–90% confluent (for ZNF692 overexpression), the transfection procedure was performed using Lipofectamine 3000 (Thermo, Massachusetts, USA) according to the manufacturer's instructions.

### siRNA and plasmid construction

The ZNF692 siRNAs were designed and synthesized by GenePharma (Shanghai, China). A total of three siRNAs were used to knock down ZNF692 expression. The sequences of the targeted siRNAs (si1 to si3) and the negative control siRNA (NC) were as follows: si1-sense: GGAAGUCUUUCAACUUUAAGATT, si2-sense: GGAUGAGGACACUGCACAAAUTT, si3-sense: GGAGCUUGCAGAUUUGGAAUCTT, NC-sense: UUCUCCGAACGUGUCACGUTT. The ZNF692plasmid containing a flag tag was constructed by Sunya (Fuzhou, China) using a gene synthesis method. The cDNA sequence of ZNF692 was synthesized according to CDS sequences of NCBI Reference Sequence: NM_001136036.3 and was inserted into pcDNA3.1-flag backbone by biosune company (http://www.biosune.com/).

### Quantitative real-time PCR (qPCR) assay

1 μg total RNA was extracted using TransZol Up kit (Transgen, Beijing, China), and reverse-transcribed into cDNA using EasyScript® One-Step gDNA Removal and cDNA Synthesis SuperMix (transgen, Beijing, China) cDNA synthesis kit. Briefly, 1 μg total RNA and 1 nmol random hexamers primers were mixed and incubated at 65 ℃ for 5 min. After incubation, the RNA and primers mixture were put on ice immediately. Then, enzyme and buffer were added to the RNA mixture and ddH_2_O was added to makeup total volume to 20 μl. The cDNAs were synthesized at 42 ℃ for 30 min and the enzyme was then inactivated at 85 ℃ for 5 s. cDNAs were quantified using Taq Pro Universal SYBR qPCR Master Mix (Vazyme, Nanjing, Chian) and a QuantStudio 3 Real-Time PCR System (ABI, California, USA) with the beta-actin (ACTB) as the reference. Gene-specific primer for qPCR were designed using NCBI “pink primer” program under default parameters with two changes: (1) primer mast span one exon-exon junction; (2) PCR product size 80 bp-300 bp. The sequences of the primer pairs were listed in Table 1. The qPCR program as following: 95 ℃ for 3min; 40 temperature cycles: 95 ℃ for 30 s,60 ℃ for 20 s,72 ℃ for 20 s; 25 ℃ to 95 ℃ by 0.1 ℃/sec for melting curve. The fluorescence signal was collected at 72 ℃ at temperature cycles stage and consecutive collected at melting curve stage.

**Table 1:** The qPCR primer pairs used in this study

Target Names	Ref sequences (NCBI accession number:)	Forward sequences (5’ to 3’)	Reverse sequences (5’ to 3’)
ACTB	NM_001101.5	CACAGAGCCTCGCCTTTGC	CCATCACGCCCTGGTGC
ZNF692	NM_001136036.3	TACCAGCACATCCACCAGAA	CGCAGAACTCACAGATGTAGTC
CDK3	NM_001258.4	TCGCTGCTCAAGGAACTGAAGC	GTCCTGGCTGAGGAACTCAAAC
G3BP2	NM_203505.3 (all isoforms)	GAGCTGAAACCACAAGTGGAGG	GGTCACTGAAGCCCAGGAGAAA
TM9SF2	NM_004800.3	CCTCCAAGAAAAGGGATGCTGC	ACAGGACCACAGCACACGTCAT

### Western blot (WB) assay

The cells were lysed with Radio immunoprecipitation assay lysis buffer (RIPA) (Pierce, 89,900), supplemented with a protease inhibitor ‘cocktail’ (Beyotime, Beijing, China). Bicinchoninic acid assay (Pierce, Illinois, USA) was used to measure protein concentrations. The protein was separated on 10% polyacrylamide gel (PAGE) and transferred to 0.45 μm nitrocellulose (NC) filter membrane (Millipore, Massachusetts, USA). Then the NC membrane was blocked with 10% nonfat powered milk (Amresco, Washington, USA) 1 h at room temperature and then hybridized with primary antibodies (ZNF692, Bioss, Beijing, China, Cat: bs-4360R; GAPDH, Beyotime, Beijing, China, Cat: AG0122) for overnight at 4 ℃ in blocking buffer. The protein-antibody complexes were incubated with peroxidase-conjugated secondary antibodies (Beyotime, Beijing, China, Cat: A0208) and were detected using enhanced chemiluminescence (Pierce, Illinois, USA).

### Cell proliferation and migration assays

ccRCC cells were cultured and transfected as described above. Forty-eight hours after transfection, cell proliferation, apoptosis, and migration assays were performed. For the cell proliferation assay, transfected cells were seeded into a 96-well plate at 10,000 cells per well, with 8 wells for each condition (siRNA vs. NC). After 24 h, a CCK8 assay was used to test cell proliferation. Cell migration was tested using a wound healing assay and a transwell assay. For the wound healing assay, 1.5 × 10^5^ cells were seeded into a 24-well plate and cultured for 12–18 h. A wound was made using a white Corning pipette tip, and the wells were washed with PBS to remove cell debris and maintained with DMEM without FBS. Twenty-four hours later, the wound was photographed with a CCD camera connected to an inverted microscope (Motic, Xiamen, China), and the wound area was analyzed by ImageJ. The transwell assay was performed using transwell inserts (Corning, 3422, New York, USA). A total of 50,000 cells were seeded into the upper inserts in 100 μl DMEM plus 1% bovine serum albumin (BSA). The cells in the inserts were cultured in 24-well plates containing 600 μl DMEM supplemented with 10% fetal bovine serum (PAN-biotech, Adenbach, Germany), 100 U/ml penicillin and 100 µg/ml streptomycin. After 24 h of incubation, the inserts were washed two times with PBS, and the attached cells were fixed with 100% methanol for 10 min. Then, the inserts were washed three times with PBS, and the cells were stained using 0.1% crystal violet for at least 1 h at room temperature. Then, the inserts were photographed with a CCD camera connected to an inverted microscope, and the cells were counted using ImageJ.

### Statistical analysis

Statistical analysis of tumor gene expression levels across clinicopathological feature subgroups was carried out using the UALCAN web tool. Comparisons of gene expression levels and IHC staining between tumor and adjacent normal tissues were performed using a paired t-test, otherwise unpaired t-tests were used. One-way ANOVA followed by a Turkey test was used to perform multiple comparison. *p* < 0.05 was set as the criterion for statistical significance. Additionally, KM survival plots with log-rank *p* values, hazard ratios (HRs), and 95% confidence intervals (CIs) were generated using the GEPIA web tool. *p* <0.05 indicated a statistically significant difference in OS.

## Results

### The prognostic role of ZNF692 in ccRCC

**Fig. 1:**
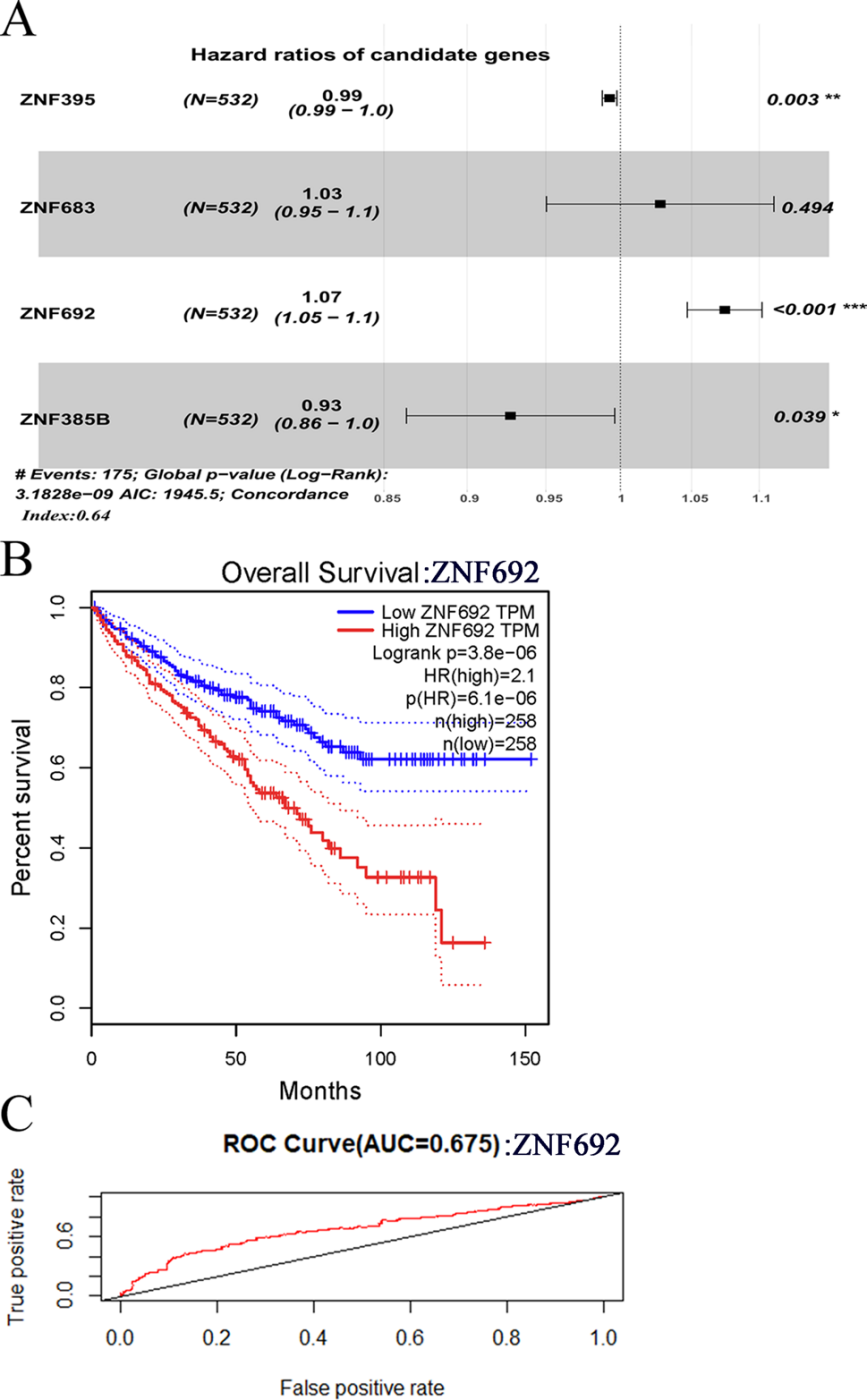
The prognostic value of ZNF692 expression in ccRCC. Analysis the prognostic value of ZNF692 using TCGA KIRC datasets. **A** The results of multivariate analysis using the LASSO–Cox regression method. **B** Effect of ZNF692 expression on the overall survival of patients with clear cell renal cell carcinoma. KM plots were generated using GEPIA (http://gepia.cancer-pku.cn/detail.php, 18, July 2022) using TCGA RNA-seq datasets (n = 533). **C** Receiver operating characteristic (ROC) curve of the overall survival of patients with high and low ZNF692 expression. The AUC was 0.675 with *p* < 0.05

To analyze the prognostic role of ZNF family genes in ccRCC, we identified the ZNF family genes that were differentially expressed in KIRC from GEPIA and evaluated the potential prognostic value of those genes using LASSO–Cox regression analysis. As shown in Fig. 1A, ZNF692 was the most promising gene for further investigation. We performed Kaplan‒Meier survival analysis using the GEPIA web tool. The results showed that higher ZNF692 mRNA expression was associated with poorer prognosis in patients with ccRCC (HR, 2.1; log-rank *p *= 3.8e−06; Fig. 1B). The mean survival time was 54.17 months in the low-expression group (lower quartile) compared to 31.53 months in the high-expression group (upper quartile). To validate the KM survival analysis results, we performed receiver operating characteristic (ROC) analysis using the “survival” package in R. As shown in Fig. 1C, the AUC was 0.675 with *1*< 0.05, suggesting that ZNF692 may be a possible prognostic factor.

### ZNF692 expression was associated with the clinicopathological features of ccRCC

**Fig. 2:**
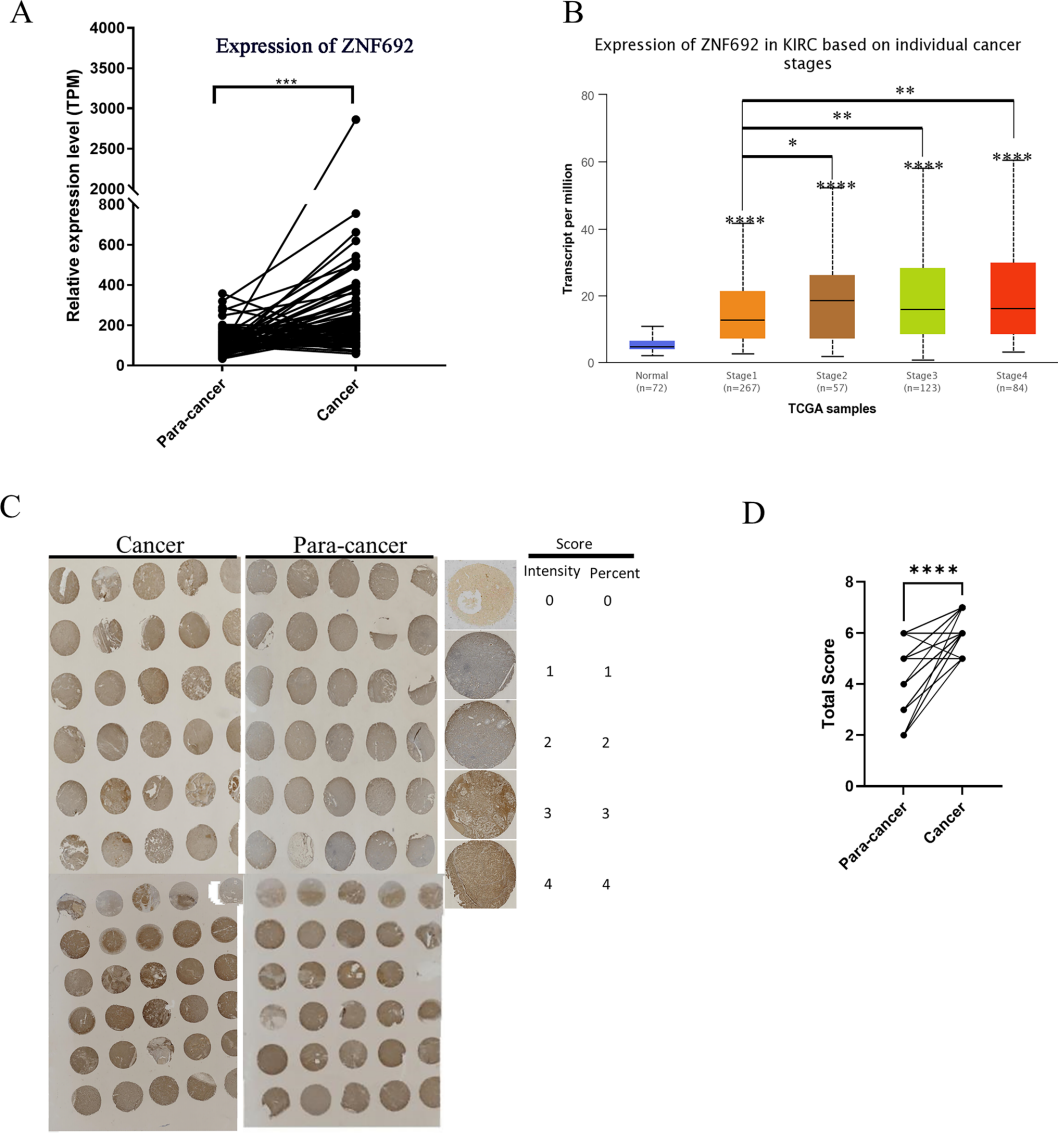
ZNF692 transcript level in clear cell renal cell carcinoma. Using TCGA ccRCC RNA-seq data, we analyzed the expression of ZNR692 in the ccRCC cohort (**A**, **B**). Using an in-house-constructed tissue microarray, we analyzed the expression of ZNF692 in ccRCC tumor tissues and tumor-adjacent tissues by IHC staining (**C, D**). **A** The expression value was calculated by the TNMplot web tool (www.tnmplot.com, 18 July 2022) using TCGA RNA-seq datasets with available data for tumor and adjacent normal tissues. The plots were generated using GraphPad Prism (V7). The ZNF692 transcript level was significantly increased (fold change = 3.02) in tumors compared with adjacent normal tissues (n = 72). **B** ZNF692 mRNA expression across histological stages of ccRCC. The expression of ZNF692 was significantly upregulated in tissues of all tumor stages relative to normal tissues. The protein expression of ZNF692 was confirmed using the Human Protein Atlas website (https://www.proteinatlas.org). **C** IHC staining of ccRCC cancer tissue (showing the results of half of the samples, n = 29; total number = 58). Upper panel: IHC staining image of the ccRCC tissue microarray; lower panel: statistical results of IHC staining. **D** The statistical result of IHC. Paired t-test was used to compare IHC staining scores between tumor and adjacent normal tissues. * indicates *P* < 0.05; ** indicates *P* < 0.01; *** indicates *P* < 0.001; **** indicates *P* < 0.0001

To investigate the role of ZNF692 in ccRCC, we analyzed expression profiles using the TCGA KIRC dataset of RNA-seq data for paired tumor and adjacent normal tissues. As shown in Fig. 2A, the expression of ZNF692 mRNA was significantly higher in the ccRCC tumor tissues than in the paired adjacent normal tissues (*p* < 0.001). To characterize the expression of ZNF692 in ccRCC, we examined the correlations between the expression of ZNF692 and the clinicopathological features of ccRCC. ZNF692 remained upregulated across discrete cancer stages (Fig. 2B), with stage 2, 3, and 4 tissues having higher levels of ZNF692 transcripts than stage 1 and normal tissues. Although there were no significant differences in ZNF692 expression level among stages 2, 3 and 4, there was an increase in the expression level of ZNF692 as the disease progressed (Table 2). To further explore and confirm the role of ZNF692 in ccRCC, we collected 58 pairs of cancer and paracancer samples from ccRCC patients (the patient information is listed in Table SI) and performed IHC analysis using an anti-ZNF692 antibody. Consistent with the mRNA expression data, the IHC results showed that the expression of ZNF692 was higher in tumor tissues than in paracancerous tissues (Fig. 2C, Table 3). Because most of our samples (41/58) were TNM stage 2 tissues, the effect of ZNF692 on tumor stage needs further analysis using a large sample size.

**Table 2:** The results of statistical analysis of the expression levels of ZNF692 in ccRCC

Clinicopathological Features	N	Expression (Mean ± SD)	Statics
Gender			
Male	345	13.21 ± 7.958	N.S
Female	188	14.944 ± 7.913	
Race			
Caucasian	462	12.978 ± 7.958^a^	N.S
African-american	56	20.701 ± 6.477^a^	
Asian	8	20.305 ± 3.64^a^	
Age			
21–40 years	26	12.768 ± 6.52^a^	N.S
41–60 years	238	15.254 ± 7.377^a^	
62–80 years	246	12.789 ± 8.516^a^	
80–100 years	23	18.545 ± 7.695^a^	
Tumor Stage			
1	**267**	**13.381 ± 4.737**^**a**^	***P* < *0.05***
2	**57**	**19.17 ± 6.92**^**b**^	
3	**123**	**15.903 ± 8.820**^**b**^	
4	**84**	**15.952 ± 8.105**^**b**^	
Nodal Metastasis			
N0	240	14.31 ± 7.723	N.S
N1	16	19.532 ± 5.821	

N.S.: No statistical significance (*P* > 0.05). superscript a, b: The same letters indicates there is no difference between sub-group (*P* > 0.05) and the different letters indicated there is significant difference between sub-group (*P* < 0.05)

Groups of statistically significant differences were shown in bold

**Table 3:** The results of statistical analysis of the expression levels of ZNF692 in ccRCC by IHC

Clinicopathological Features		N	Statics
Gender	Male	46	N.S
	Female	12	
Age	21–40 years	6	a
	41–60 years	24	a
	61–80 years	26	a
	81–100 years	2	a
Histological Grade	1	6	a
	2	42	a
	3	8	a
	4	2	a
Nodal Metastasis	N0	58	Non
	N1	0	

N.S.: No statistical significance (*P* > 0.05); Non: Unable to perform statistical analysis for the small number of samples. a: The same latter indicates there is no difference between sub-group (*P* > 0.05)

### ZNF692 promotes ccRCC cell proliferation and migration

**Fig. 3:**
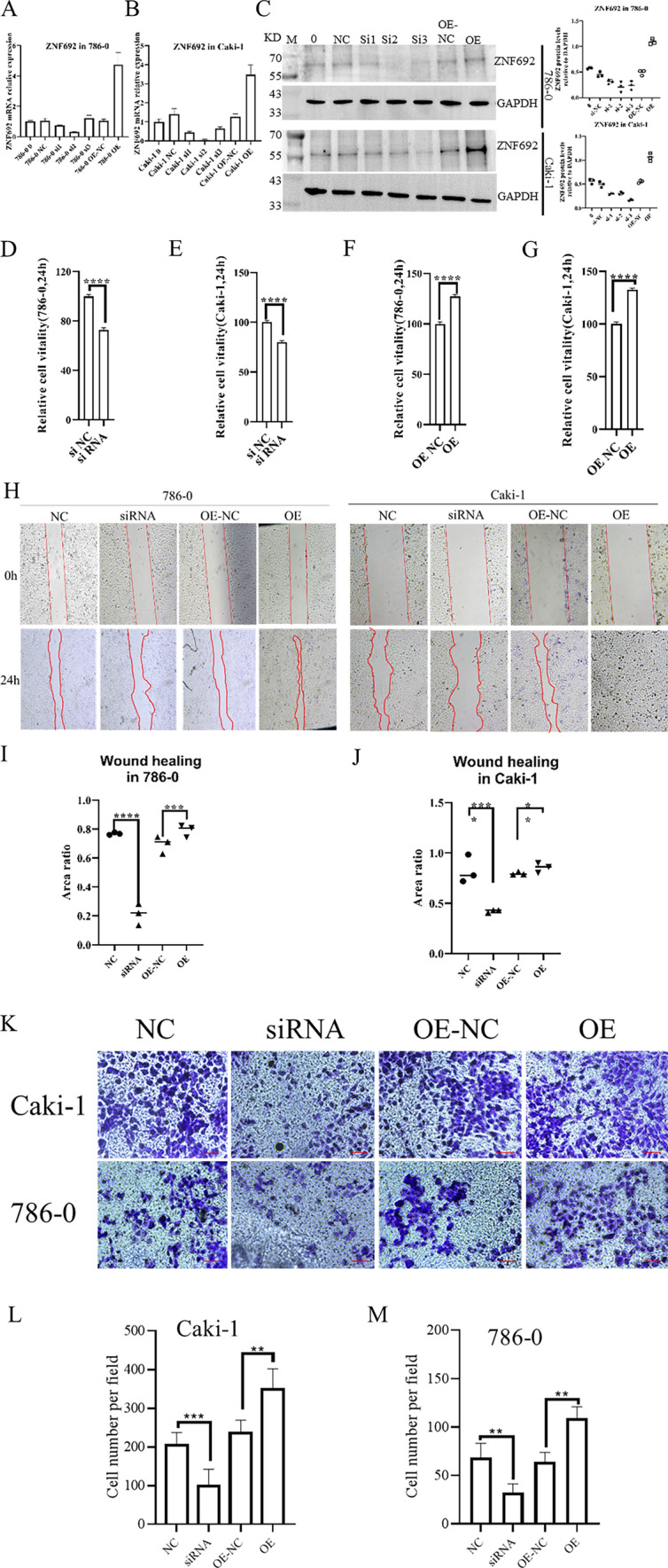
ZNF692 promoter ccRCC cells proliferation and migration. ZNF692 was Knockdown (KD) or overexpressed (OE) in ccRCC cell lines 786–0 and Caki-1. qPCR and WB were used to test the effects of KD and OE (**A** to **C**). The cell proliferation was tested using CCK8 method (**D** to **J**) and the cell migration was tested using wound healing assay (**H** to J) and transwell assay (**K** to **M**). **A** qPCR tested the effects of KD and OE in the 786–0 cell line. **B** qPCR tested the effects of KD and OE in the Caki-1 cell line. **C** WB tested the effects of KD and OE of ZNF692 in 786–0 and Caki-1 cell lines. **D** The KD of ZNF692 inhibited the migration of 786–0 cells. **E** the KD of ZNF692 inhibited the migration of Caki-1 cells. **F** The OE of ZNF692 promoted the migration of 786–0 cells. **G** The OE of ZNF692 promoted the migration of Caki-1 cells. **H** The quantification of wound healing assay in 786–0 and Caki-1 cells. **I** The quantification of wound healing assay in 786–0 cells. **J** The quantification of wound healing assay in Caki-1 cells. **K** The results of transwell assay in 786–0 and Caki-1 cell lines. The red scale bar represents 100 μm (**I**) The quantification of transwell assay in 786–0 cells. **J** The quantification of transwell assay in Caki-1 cells. All those assays were repeated for three times. * indicates *P* < 0.05; ** indicates *P* < 0.01; *** indicates *P* < 0.001; **** indicates *P* < 0.0001

To test the hypothesis that ZNF692 is an oncogene, we performed cell proliferation and migration assays using the ccRCC cell lines Caki-1 and 786–0. We used the transfection of ZNF692 plasmid to overexpress (OE) ZNF692 and used siRNA methods to knockdown (KD) the expression of ZNF692. We designed three siRNA to KD the expression of ZNF692 and used qPCR and WB to test the effects of siRNA. QPCR and WB data (Fig. 3A–C) showing that si-2 had the best effects of KD. So si-2 was used in the flowing study to KD ZNF692. As shown in Fig. 3D, E, ZNF692 KD inhibited the proliferation of ccRCC cells; in contrast, ZNF692 OE (Fig. 3F, G), promoted the proliferation of ccRCC cells. To test the role of ZNF692 in the migration of ccRCC cells, we performed wound healing assay and Transwell assay. The results of the wound healing assay showed that the migration of ccRCC cells was inhibited when ZNF692 was knocked down, while the migration of ccRCC cells was promoted when ZNF692 was overexpressed (Fig. 3H–J). Consistent with wound healing assay, the transwell assay results exhibited that the KD of ZNF692 inhibited the migration of ccRCC cells and the overexpression of ZNF693 promoted the migration of ccRCC cells (Fig. 3K–M).

### Pathway enrichment and gene ontology enrichment analyses of ZNF692-related genes

Since there are limited reports regarding ZNF692 target genes, we determined the genes whose expression correlated with ZNF692 expression and performed gene set enrichment analysis (GSEA) using those correlated genes to explore ZNF692 target genes. The “LinkFinder” and “LinkInterpreter” modules of the LinkedOmics online tool were used to identify genes whose expression was positively or negatively correlated with ZNF692 expression and were then used to perform GO biological process analysis. The positively correlated genes accounted for the majority of the correlated genes and were almost twice as numerous as the negatively correlated genes (Figure S1A). The GSEA results showed that the genes negatively coexpressed with ZNF692 were enriched in pathways associated with the immune system response or inflammation. The most enriched pathways were adaptive immune response, regulation of cytokine production, and T and B cell activation (Figure S1B, orange bars). Moreover, the genes positively coexpressed with ZNF692 were enriched in pathways associated with the energy metabolism process and protein transport and targeting (Figure S2B, yellow bars), such as mitochondrial respiratory chain complex assembly, mitochondrial gene expression, and protein transmembrane transport.

**Fig. 4:**
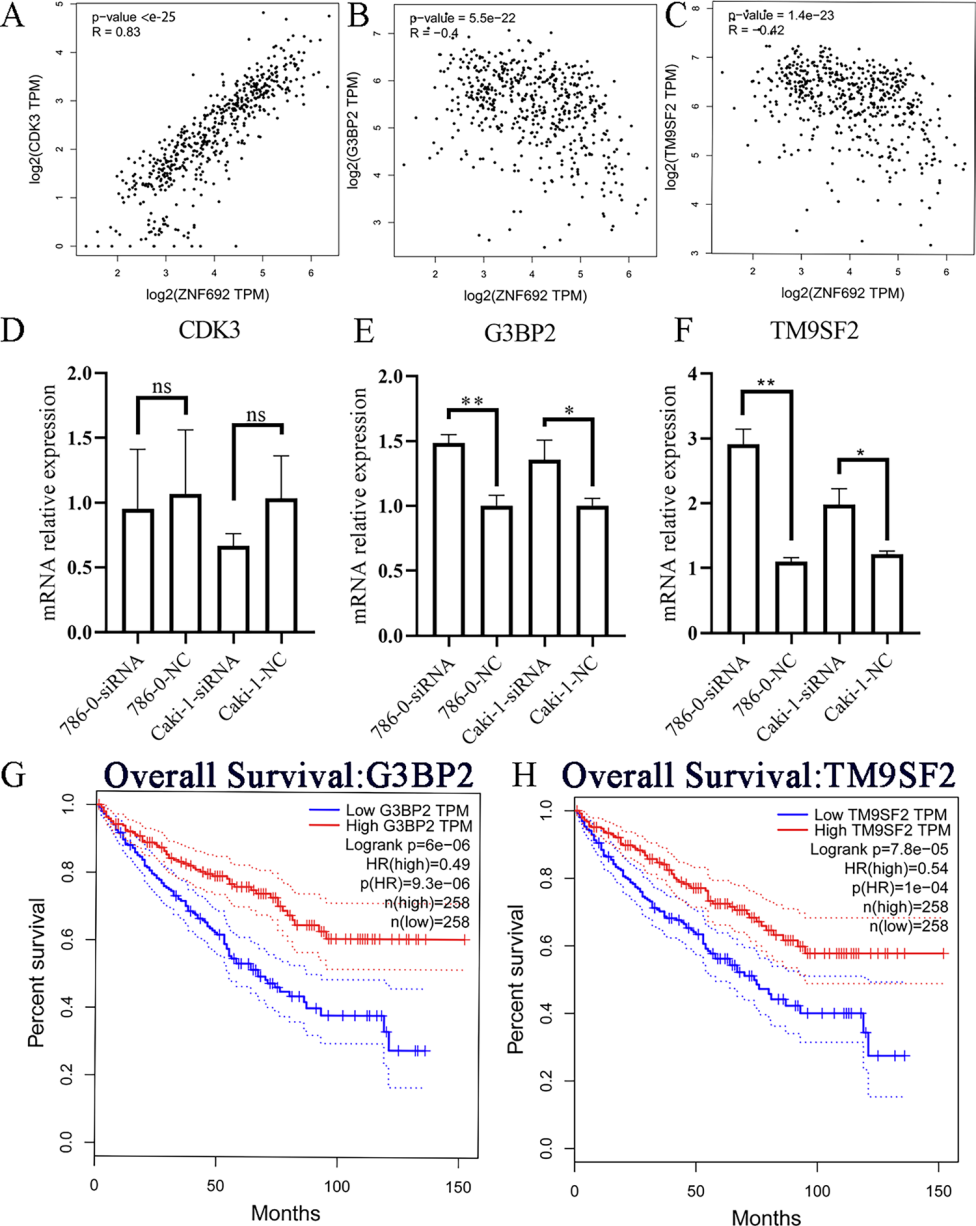
ZNF692 targeted G3BP2 and TM9SF2. The correction between ZNF692 and G3BP2 and TM9SF2 was analyzed by GEPIA web tools using TCGA KIRC dataset and tested by qPCR in ccRCC cells (**A** to **E**). The survival curve of G3BP2 and TM9SF2 in ccRCC was analyzed using GEPIA web tools (**G** and **H**). **A** The positively associated of CDK3 with ZNF692. **B** The negatively associated of G3BP2 with ZNF692. **C** The negatively associated of TM9SF2 with ZNF692. (**D** to **F**) qPCR tested the relation between ZNF692 and CDK3, G3BP2, and TM9SF2 respectively. **G** The high expression of G3BP2 indicated good prognostic in TCGA KIRC cohort. **H** The high expression of TM9SF2 indicated good prognostic in TCGA KIRC cohort. All those assays were repeated for three times

To test ZNF692 target genes, we analyzed the expression of three gene (cyclin dependent kinase 3 (CDK3), GTPase-activating protein (SH3 domain)-binding protein 2 (G3BP2), and transmembrane 9 superfamily member 2 (TM9SF2)), which involved in metabolism or cell cycle, form top 5 coexpressed genes with ZNF692 (Figure S2A). In those three genes, CDK3 was positively associated with ZNF692, G3BP2 and TM9SF2 were negatively associated with ZNF692 (Fig. 4A–C). To explore the role of ZNF692 in the regulation of the expression of CDK3, G3BP2 and TM9SF2, we knockdown the expression of ZNF692 by siRNA and the mRNA expression levels of CDK3, G3BP2 and TM9SF2 were tested using qPCR in ccRCC cell lines 786-0 and Caki-1. As showed in Fig. 4D–F, the KD of ZNF692 did not significantly change the expression of CDK3, however, the expression of G3BP2 and TM9SF2 gene was increased by KD of ZNF692. Furthermore, the prognostic analysis using GEPIA web tools showed that the high expression of G3BP2 and TM9SF2 associated with poor prognostic in the TCGA KIRC cohort (Fig. 4G–H). All those data indicated that ZNF692 negatively regulated G3BP2 and TM9SF2 expression in ccRCC.

### The expression pattern of ZNF692 in kidney tissue

**Fig. 5:**
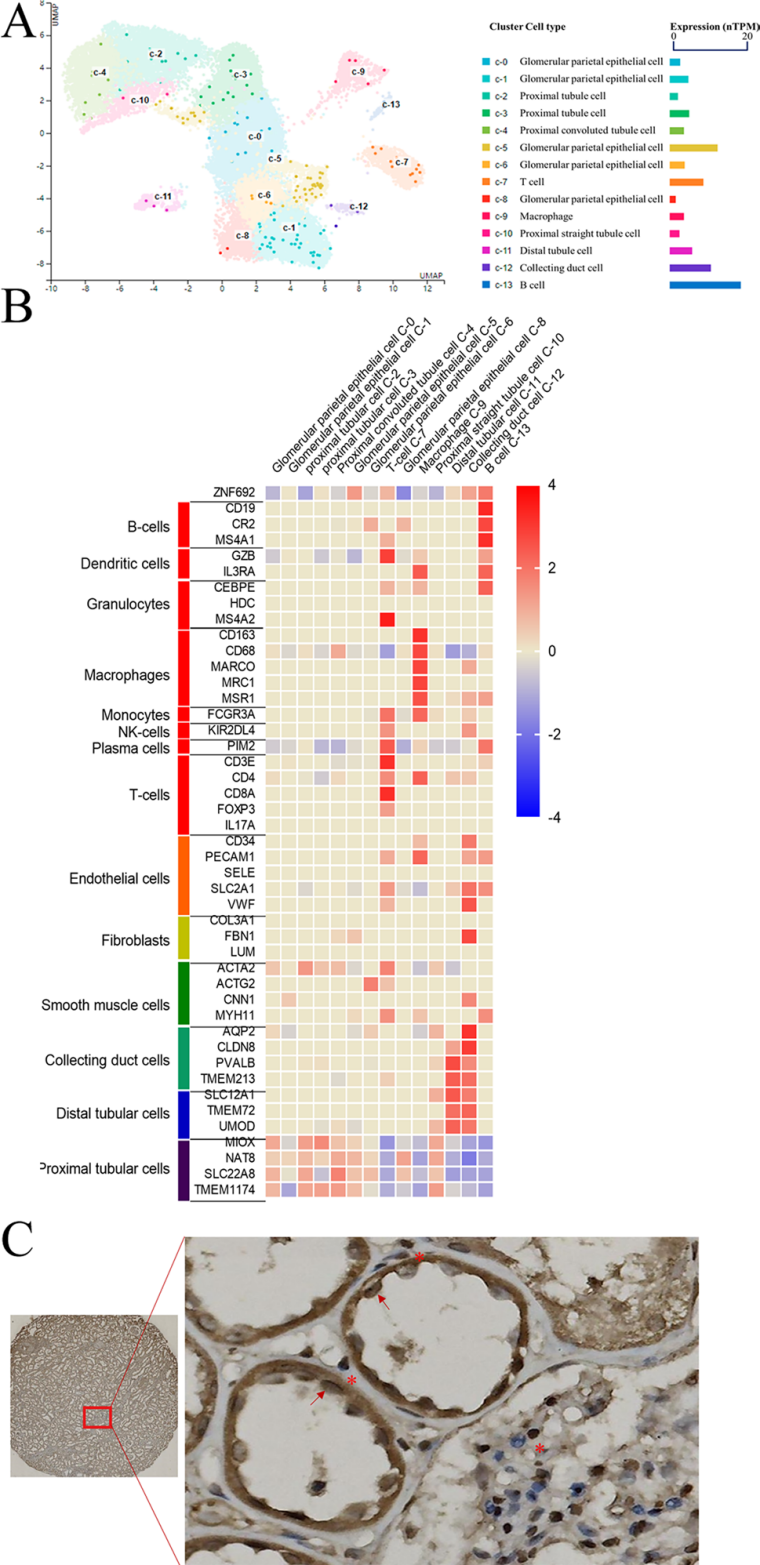
The expression pattern and distribution of ZNF692 in normal human kidney tissue. Single-cell sequencing datasets of normal human kidneys from the Human Protein Atlas website (https://www.proteinatlas.org, 18 July 2022) were used to analyze the expression pattern of ZNF692 (**A**, **B**). We performed IHC staining to analyze the distribution of ZNF692-expressing cells in tumor-adjacent tissues (**C**). **A** The UMAP cluster plot indicates the 13 types of cells in normal human kidney tissues and the cell types in which ZNF692 was expressed. **B** The heat maps show the expression of markers for each cell type and the expression of ZNF692 in each cell type. **C** The IHC staining results show the distribution of ZNF692-positive cells in normal tissue. Endothelial cells in renal tubules are labeled with a red arrow, and cells in the kidney mesenchyme are labeled with a red star

To further explore the role of ZNF692 in ccRCC, we analyzed scRNA-seq data from normal human kidney tissue using the scRNA section of The Human Protein Atlas. As shown in Fig. 5A, B, the uniform manifold approximation and projection (UMAP) plot and heatmap showed that there are 13 cell types in normal human kidney tissue: T cells, B cells, macrophages, and various types of tubular cells. T and B cells are the main immune cells expressing ZNF692, and proximal tubular cells (c-5, glomerular parietal epithelial cells) and collecting duct cells (c-12) are the main epithelial cells that express ZNF692. Our IHC results showed that ZNF692 was expressed mainly in endothelial cells of the tubules (Fig. 5C, indicated by red arrows) and cells in the mesenchyme (Fig. 5C, indicated by red stars). The expression pattern of ZNF692 in normal kidney tissue is consistent with its function inferred from the above GSEA results.

### ZNF692-expressing ccRCC tumor cells respond to ICB therapy

**Fig. 6:**
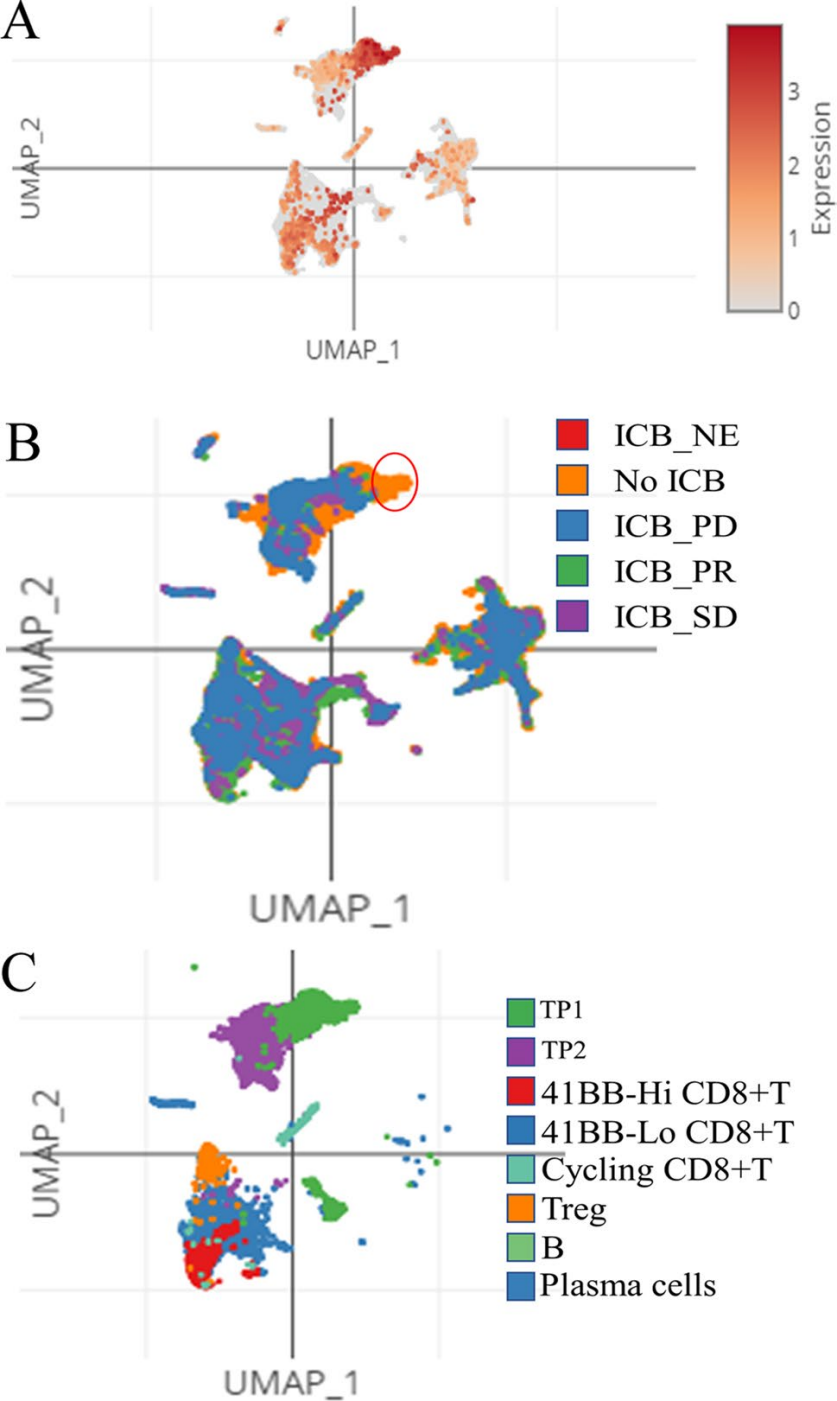
The function of ZNF692 in ccRCC patients receiving ICB therapy. Using the keyword “kidney” to search the studies section of the Single Cell Portal web tool (https://singlecell.broadinstitute.org, 18, July 2022), we targeted the study “tumor and immune reprogramming during immunotherapy in advanced renal cell carcinoma” with the accession number SCP1288 for further analysis. We analyzed the expression pattern of ZNF692 in ccRCC patients who received TKI therapy and patients who received ICB therapy. **A** The UMAP plots show the expression pattern of ZNF692 in all cell types from all samples. **B** The plots show the different expression patterns of ZNF692 between patients with ccRCC who did or did not receive ICB therapy. **C** The expression pattern of ZNF692 in the final defined cell types isolated from patients with ccRCC

Given that ZNF692 was primarily expressed in T and B immune cells, proximal tubular cells, and collecting duct cells, we investigated whether ZNF692 is involved in the response to ICB therapy in ccRCC. To assess the role of ZNF692 in the response to ICB therapy in ccRCC, we queried the Single Cell Portal database with the keyword “kidney”. We found a dataset containing 34326 cells in which the single-cell transcriptomes of cancer and immune cells isolated from patients with metastatic ccRCC before or after ICB therapy were characterized [21]. Fig. 6A shows the expression pattern of ZNF692 across all cell types. Upon exposure to ICB agents, some ZNF692-expressing cells disappeared from the data (Fig. 6B, red circle). UMAP clustering showed that tumor program 1 (TP1) tumor cells were the dominant cells expressing ZNF692 (Fig. 6C) and were responders to ICB therapy. Next, we explored the role of ZNF692 in TP1 cells using coexpression analysis and GSEA of the differentially expressed genes between TP1 and TP2 cells (the genes are listed in Table SII). The most enriched pathway of the positively correlated genes was the spliceosome pathway. Moreover, the pathway that positively regulates type I interferon production was the most enriched pathway of the genes with a negative correlation (Figure S2).

## Discussion

ccRCC is highly treatable if diagnosed at an early stage. Unfortunately, approximately 30% of patients are diagnosed with renal carcinoma in a later stage with a poor prognosis and limited treatment options [25]. Thus, there is a need to identify new markers that can be used to diagnose ccRCC as early as possible. ZNF692 is linked to the recurrence of Wilms’ tumor [12]^,^ and promotes the proliferation and invasion of cervical cancer cells [16]. ZNF692 associates with the prognosis and proliferation of ccRCC [13, 14]. In addition, ZNF692 may serve as a potential biomarker associated with immune infiltration in hepatocellular carcinoma [17]. However, the role of ZNF692 in ccRCC is largely unknown. In this study, we found that ZNF692 was significantly upregulated in tumor tissues compared with adjacent normal tissues (Fig. 2), with stage 2, 3, and 4 tissues having higher ZNF692 expression levels than stage 1 tissues. Consistent with the findings of Xu et al. [13, 14], higher mRNA expression of ZNF692 was linked with poor overall survival in ccRCC patients (Fig. 1B).

786-0 is a ccRCC cell line derived from a primary clear cell adenocarcinoma (https://www.atcc.org/products/crl-1932) and Caki-1 derived from a primary clear cell adenocarcinoma with metastasis (https://www.atcc.org/products/htb-46#detailed-product-information). So, we used those two cells to study the role of ZNF692 in the migration of ccRCC cells. Our wound healing and Transwell assay showed that the migration potential of Caki-1 was higher than that of 786-0. In vitro cells experiment shown that ZNF692 promoted the proliferation and migration of ccRCC cells (Fig. 3). However, due to the limitation of experimental conditions, we unable to perform in vivo experiments to confirm the function of ZNF692 in ccRCC. This is a limitation of this study. T cells, B cells, proximal tubular cells, and collecting tubule cells are the dominant cell types in normal kidney tissue where ZNF692 is expressed. In addition, the expression of ZNF692 in tumor program 1 (TP1) tumor-like cells dramatically changed after patients received ICB therapy, with loss of ZNF692 expression in TP1 cells. These data strongly suggest that ZNF692 could function as an oncogene and that ZNF692 may serve as an indicator of the response to ICB therapy.

Due to the limited data on ZNF692 target genes, we firstly explored the genes co-expressed with ZNF692 and performed GSEA to analyze the function of ZNF692 in ccRCC (Figure S2). Genes positively coexpressed with ZNF692 were involved in pathways such as energy metabolism and mitochondrial regulation. Renal cell carcinoma (RCC) is essentially a metabolic disease characterized by a reprogramming of energetic metabolism [26–29]. The metabolic reprogramming covers different processes such as fatty acid metabolism [30, 31] and glycolysis with partitioned metabolic flux through glycolysis and impaired mitochondrial bioenergetics and OXPhox [32–35]. The co-expression analysis and GSEA results showed that the top 3 pathways were mitochondrial respiratory chain complex assembly, NADH dehydrogenase complex assembly, and mitochondrial gene expression, suggesting that the target genes of ZNF692 may be involved in the regulation of metabolic homeostasis.

However, the genes negatively coexpressed with ZNF692 were involved in pathways such as the immune response, cytokine production, and T and B cell activation. In the last few years, several studies have showed that the activation of specific metabolic pathway have a role in regulating angiogenesis and inflammatory signatures to reprogram tumor microenvironment [36, 37]. Our results suggests that ZNF692 may govern immune homeostasis in the tumor microenvironment of ccRCC.

The Chip-seq and qPCR data showed that ZNF692 may target G3BP2 and TM9SF2 to function in ccRCC cell. ZNF692 KD increased the expression of negatively associated genes G3BP2 and TM9SF2 (Fig. 4E, F). In addition, the expression of G3BP2 and TM9SF2 were lower in ccRCC compared with normal tissues. G3BP2 was first identified as an androgen-responsive gene, core components that contributes to stress granules (SGs) assembly and RNA metabolism [38]. Increased G3BP2 promoted the growth of cancer cells and high expression level of G3BP2 was associated with poor prognostic in Head and Neck squamous cell carcinoma, prostate cancer, breast, and lung cancer [39–42]. However, we found that the high expression level G3BP2 was associated with better prognostic in ccRCC. TM9SF2 is a member of the TM9SF family which has four reported members in mammals (TM9SF1-4) with characteristic of nine transmembrane domains [43]. TM9SF2 may regulate the biosynthesis of glycosphingolipids by regulates the subcellular localization of globotriaosylceramide synthase [43].TM9SF2 may be an oncogene and a prognostic marker with high expression associated with poor prognostic in pancreatic adenocarcinoma and colorectal cancer [44, 45]. But we found that in TCGA ccRCC cohort TM9SF2 was associated with good prognostic. This suggests the difference of the role of the same gene in different cancer type and large clinical samples are needed to confirm the role of G3BP2 and TM9SF2 in ccRCC.

Single-cell RNA sequencing is capable of resolving the complexity of tissue and tracing cell lineage differentiation. Since ZNF692 is involved in both energy metabolism and immune regulation in ccRCC, we next explored single-cell RNA-seq data to analyze the expression pattern of ZNF692. ZNF692 was expressed in both kidney-resident immune cells and kidney tissue cells. Specifically, B cells and T cells are the main immune cells that express ZNF692, and proximal tubular cells and collecting tubule cells are the main kidney tissue cells that express ZNF692. The IHC results showed similar patterns of ZNF692 expression in kidney-resident immune cells and kidney tissue cells identified by mRNA expression analysis. The expression pattern of ZNF692 in normal kidney tissue was consistent with the GSEA results in ccRCC cells, suggesting that ZNF692 is involved in the immune response and mitochondrial regulation.

Abundant infiltration of immune cells, particularly T cells, is one of the characteristics of the ccRCC tumor microenvironment [46–49]. Features of the tumor microenvironment heavily affect disease biology and may affect responses to systemic therapy especially immunotherapy [50–53]. In this context, immunotherapy for ccRCC has progressed considerably over the past decade, with new options ranging from cytokine treatments [54] to targeted therapies such as ICB and VEGF tyrosine kinase inhibitors (TKIs) [55, 56]. Upon treatment with PD-1 inhibitors, the 4-1BB-Lo progenitor exhaustion signature of CD8^+^ T cells was found to be highly enriched, but the other subtype of CD8^+^ T cells was largely unchanged [21]. Considering the expression of ZNF692 in T and B cells in normal kidney tissue, we hypothesized the immunotherapeutic role of ZNF692 in ccRCC.

The tumor-like cell population was composed of two subtypes of cells, TP1 and TP2 [20]. The TP1 subtype, which exhibited robust upregulation of nectin-2 after treatment, expressed a signature associated with improved survival, while the TP2 subtype expressed several checkpoint molecules. Cluster analysis showed that the main cell type with high ZNF692 expression was the TP1 subtype (Fig. 6C). Our GO analysis using the correlated genes differentially expressed in TP1 cells also showed that ZNF692 was involved in the activity of T and B cells and type I interferon production (Figure S1 and Figure S2). Collectively, these data suggest that tumor cells with high ZNF692 expression may be the primary responders to ICB therapy, but further experimental and clinical verification is needed.

In summary, ZNF692, a member of the Krüppel C2H2‑type zinc finger protein family, is differentially expressed in several tumors. In ccRCC, the expression of ZNF692 was significantly higher in tumor tissue than in adjacent normal tissue, and the expression of this gene was associated with more advanced cancer stage and poor prognosis. In ccRCC cell lines, 786-0 and Caki-1, ZNF692 promoted the cells' proliferation and migration by target G3BP2 and TM9SF2. In addition, ZNF692 was expressed in T cells, B cells, and renal tubular cells. However, the expression pattern of ZNF692 in tumor-like cells was dramatically altered after ICB therapy. Therefore, ZNF692 may represent a predictive risk factor for oncogenesis and a biomarker for the response to immunotherapy in ccRCC, although additional large-scale validation is required. Given that the transcriptional targets and regulators of ZNF692 are largely unknown, comprehensive analysis and validation are needed to confirm the targets of ZNF692, particularly in ccRCC.

### Supplementary Information

Supplementary file 1

Supplementary file 2 (TIF 1717 KB)

Supplementary file 3 (DOCX 145 KB)

Supplementary file 4 (XLSX 14 KB)

Supplementary file 5 (XLSX 18 KB)

## Data Availability

All the data used in this study are available publicly in the TCGA database under the project KIRC and in the Single Cell Portal under the study name “Tumor and immune reprogramming during immunotherapy in advanced renal cell carcinoma” (10.1016/j.ccell.2021.02.015, ref: 23). The data generated or analyzed in the current study are available from the corresponding author upon reasonable request.
