# Supplementary figures and images for: ZNF692 promotes the migration and response to immunotherapy of clear cell renal cell carcinoma cells by targeting metabolic pathway
Source: Discov Oncol. 2024 May 12;15:158. doi: 10.1007/s12672-024-01005-0 (PMC11089031; doi:10.1007/s12672-024-01005-0)

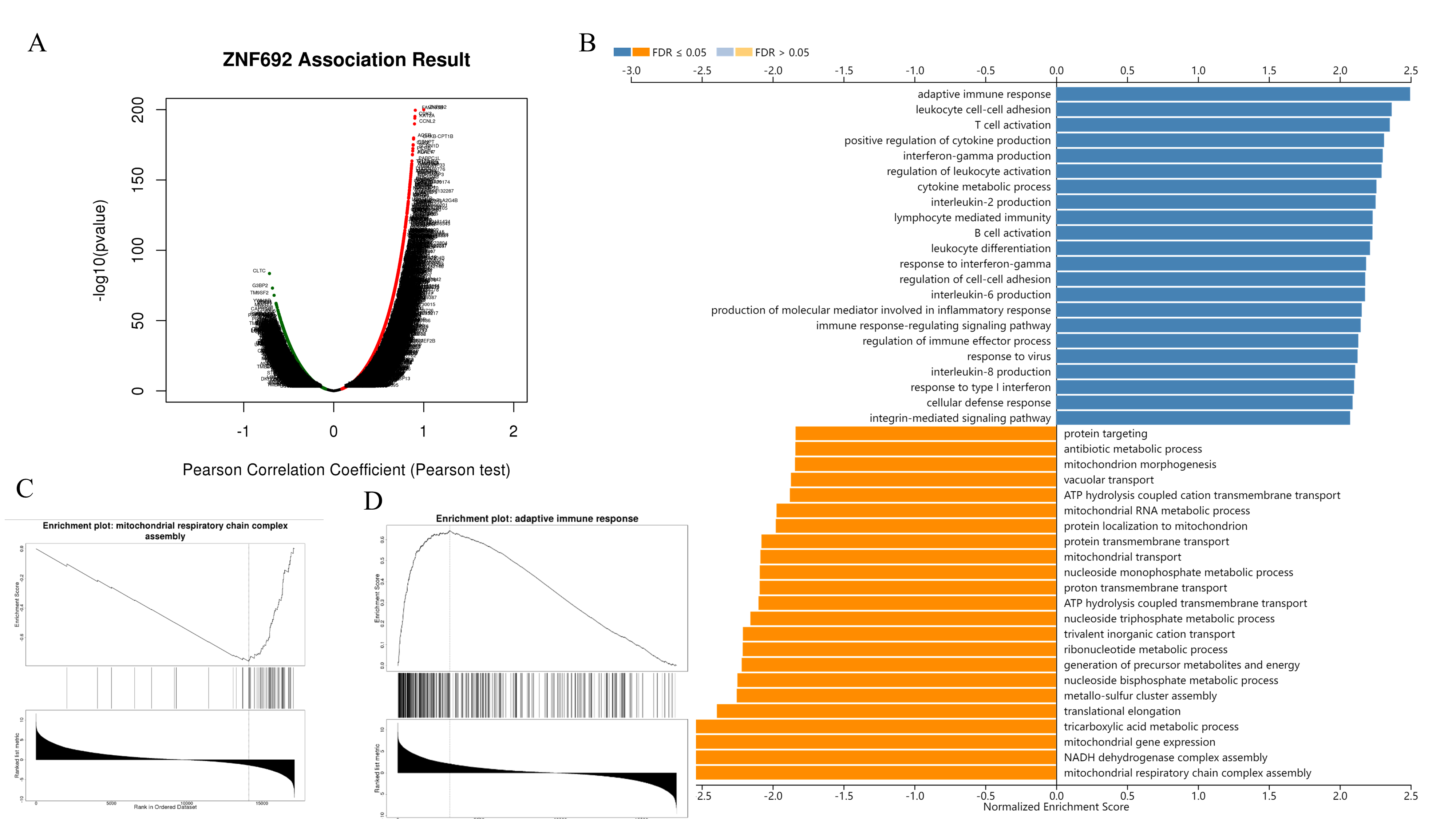

Supplement: Supplementary file 1 [file 12672_2024_1005_MOESM1_ESM.tif]

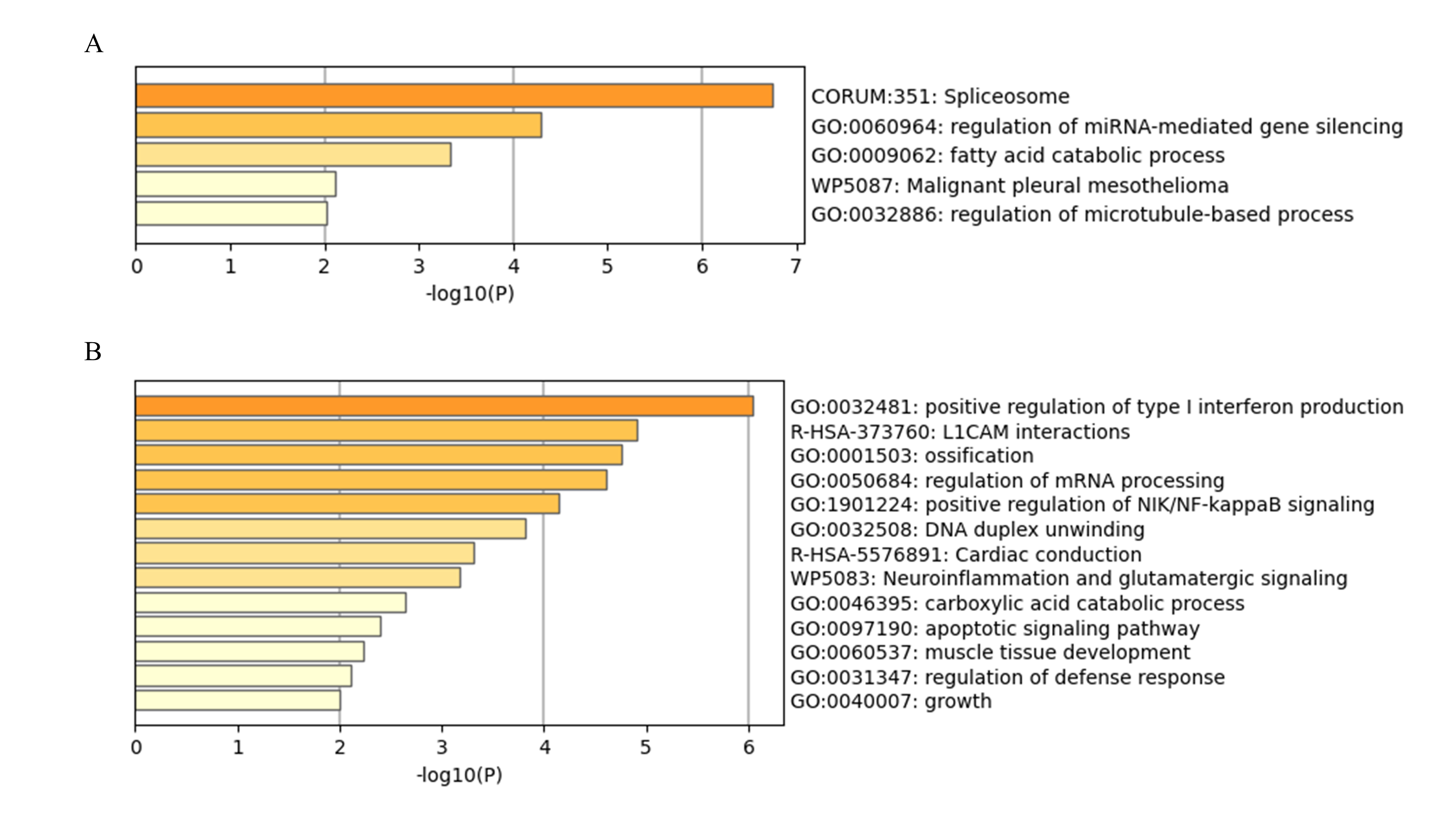

Supplement: Supplementary file 2 — (TIF 1717 KB) [file 12672_2024_1005_MOESM2_ESM.tif]
