# Supplementary material for: ZNF692 promotes the migration and response to immunotherapy of clear cell renal cell carcinoma cells by targeting metabolic pathway
Source: Discov Oncol. 2024 May 12;15:158. doi: 10.1007/s12672-024-01005-0 (PMC11089031; doi:10.1007/s12672-024-01005-0)
Supplement: Supplementary file 3 — (DOCX 145 KB) [file 12672_2024_1005_MOESM3_ESM.docx]

Supplementary Figures and Tables legends

Orignioal WB files (corresponding to Figure 3C in main manuscript)

The cells were lysed with Radio immunoprecipitation assay lysis buffer (RIPA), supplemented with a protease inhibitor ‘cocktail’. The protein was separated on 10% polyacrylamide gel (PAGE) and transferred to 0.45μm nitrocellulose (NC) filter membrane. Then the NC membrane was blocked with 10% nonfat powered milk 1 h at room temperature and then hybridized with primary antibodies (ZNF692, Bioss,Beijing, China,Cat: bs-4360R ; GAPDH, Beyotime, Beijing, China, Cat: AG0122) for overnight at 4 ℃ in blocking buffer. The protein-antibody complexes were incubated with peroxidase-conjugated secondary antibodies (Beyotime, Beijing, China, Cat: A0208) and were detected using enhanced chemiluminescence (Pierce, Illinois, USA).

gel images 1.


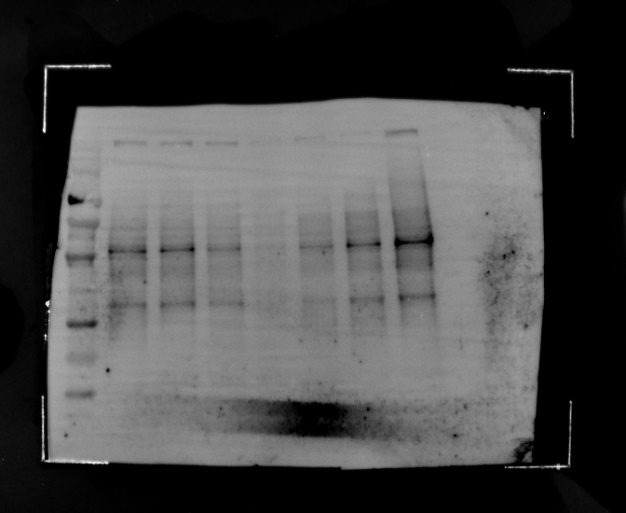


WB blots of ZNF692 (in reference to Figure 3C up-panel ZNF692) in 786-0 cell line. There are 8 lanes (from left to right): Blank-positive, siRNA-NC, siRNA-1, siRNA-2, siRNA-3 OE-NC, OE, Blank-negtive (for ZNF692).

gel images 2.





WB blots of GAPDH (used as reference; in reference to Figure 3C up-panel GAPDH) in 786-0 cell line. There are 8 lanes (from left to right): Blank-positive, siRNA-NC, siRNA-1, siRNA-2, siRNA-3 OE-NC, OE, Blank-negtive (for ZNF692).

gel images 3.


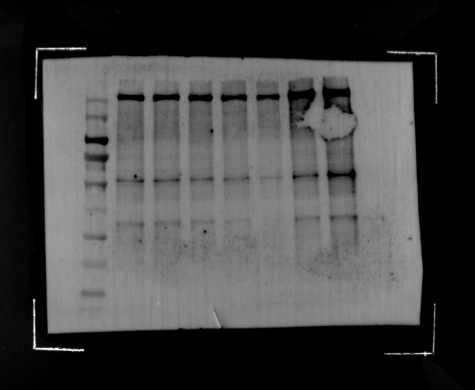


WB blots of ZNF692 (in reference to Figure 3C down-panel ZNF692) in Caki-1 cell line. There are 8 lanes (from left to right): Blank-positive, siRNA-NC, siRNA-1, siRNA-2, siRNA-3 OE-NC, OE, Blank-negtive (for ZNF692).

gel images 4.





WB blots of GAPDH (used as reference; in reference to Figure 3C down-panel GAPDH)

in Caki-1 cell line. There are 8 lanes (from left to right): Blank-positive, siRNA-NC, siRNA-1, siRNA-2, siRNA-3 OE-NC, OE, Blank-negtive (for ZNF692).
